# An Interventional strategy of physical activity promotion for reduction of menopause symptoms

**DOI:** 10.34172/hpp.2020.57

**Published:** 2020-11-07

**Authors:** Zeinab Javadivala, Hamid Allahverdipour, Mohammad Asghari Jafarabadi, Azita Emami

**Affiliations:** ^1^Department of Health Education & Promotion, Tabriz University of Medical Sciences, Tabriz, Iran; ^2^Research Center for Psychiatry and Behavioral Sciences, Tabriz University of Medical Sciences, Tabriz, Iran; ^3^Department of Health Education & Promotion, Tabriz University of Medical Sciences, Tabriz, Iran; ^4^Medical Education Research Center, Faculty of Health, Tabriz University of Medical Sciences, Tabriz, Iran; ^5^Dean, University of Washington School of Nursing, Seattle, Washington, USA

**Keywords:** Exercise, Menopause, Health Promotion, Social network

## Abstract

**Background:** Physical activity (PA) programs are inexpensive, non-pharmaceutical and universally accessible options with demonstrated efficacy in reducing menopausal symptoms. The purpose of this study was to determine the effectiveness of a behavioral strategy for initiating and sustaining PA with the hope to reduce or eliminate menopausal symptoms.

**Methods** : Menopausal and perimenopausal women (n=190) were randomly assigned to intervention (n=95) and non-intervention (n=95) groups using a random-numbers table. The intervention group consisted of 18 neighborhood network subgroups, each consisting of five to six women known to one another. They participated in a 12-week regular PA program, augmented by eight interactive group education and discussion sessions. The Menopause Rating Scale (MRS) self-report instrument was used to determine perceived severity of menopausal symptoms.

**Results:** The intervention group showed a significant reduction in the frequency and severity of menopausal symptoms (P < 0.001). Those whose symptoms rated severe/very severe for hot flushes were reduced from 30.1% to 11.8%. Also, participants whose sleep problems and joint discomfort rated severe/very severe declined from 28% to 6.5% and joint discomfort rated severe or very severe was reduced from 52.7% to 4.4%, respectively. Conversely in the nonintervention group, hot flushes, sleep problems and joint problems got significantly worse(P < 0.05).

**Conclusion:** Implementing educational program that increases awareness of PA benefits in combination with existing neighborhood networks that facilitate communication and cooperation may increase PA levels and decrease menopausal symptoms. Such networks offer alow-cost means of improving quality of life (QOL) for perimenopausal and menopausal women.

## Background

In many parts of the developing world, the use of sophisticated medical resources is limited by both cost and geographic accessibility. This makes strategies that respond to these challenges particularly valuable, especially for conditions that affect a substantial portion of the population such as adverse symptom resulting from menopause.^1^

Much of the female population experiences menopausal symptoms severe enough to have an adverse impact on physical and mental health.^1^ These quality of life (QOL) symptoms include hot flashes, vaginal dryness, joint pain, fatigue, poor sleep, irritability, and depression.^2^ Many women experience an idiosyncratic cluster of such symptoms.^3^

Menopausal hormone therapy (MHT) can effectively counter these symptoms, lessening or eliminating 80% to 90% of the symptoms compared with other medical interventions.^4^ However, such therapy requires medical access and economic means that are scarce in developing countries—particularly in rural areas. Even when patients have access to a physician, there is reluctance on the part of many doctors to prescribe MHT as a first-line treatment due to increased risk of heart disease, breast cancer, endometrial cancer, and thromboembolic events such as stroke.^5,6^ In Iran, MHT usage ranges from 4.5% to 12.5% of the menopausal women.^7,8^

One study found that menopausal women in Iran scored higher on menopausal symptoms than European woman. About 72% of the women in Iran did not consult a physician and preferred to accept menopausal signs and symptoms as inevitable and unchangeable or as something they just have to live with.^9^

Shaikh estimates that, “more than 70% of the developing world’s population still depends on the complementary and alternative systems of medicine. Cultural beliefs and practices often lead to self-care or home remedies in rural areas and consultation with traditional healers.”^10^ Even in an urbanized First World area, 22.1% of the women in a population-based survey reported using one of eight alternative therapies to ameliorate menopause symptoms.^11^

Physical activity (PA) is often not considered when discussing “complementary and alternative medicine,” yet its broad spectrum of health benefits is well documented.^12,13^ “We confirm that there is irrefutable evidence of the effectiveness of regular PA in the primary and secondary prevention of several chronic diseases (e.g., cardiovascular disease, diabetes, cancer, hypertension, obesity, depression and osteoporosis) and premature death.”^14-16^

Multiple studies have also demonstrated the positive impact of PA on menopausal symptoms, with Elavsky and McAuley concluding that, “The results indicated that walking and yoga were effective in enhancing positive affect and menopause-related QOL and reducing negative affect. Women who experienced decreases in menopausal symptoms across the trial also experienced improvements in all positive mental health and QOL outcomes and reductions in negative mental health outcomes.”^17^ A Swedish study reported that only 5% of women who exercised regularly experienced “severe” hot flashes, compared to a rate of 15% in sedentary women.^18^

While menopausal symptoms have a substantial impact on many women’s QOL globally, and although PA has been shown to reduce or eliminate such symptoms, there has been little to no research on ways to encourage and sustain PA in populations where sedentary behavior is the norm. In fact, “prescribing” more PA has proved one of the orders with which patients are least compliant. Blair is not alone in calling PA “the biggest public health problem of the 21^st^ century.”^19^

Without evidence-based knowledge about the behavioral and social components of PA, effective use of PA in lieu of MHT will remain an elusive goal. The purpose of this study was to determine the effectiveness of a behavioral strategy for initiating and sustaining PA that may reduce or eliminate menopausal symptoms among women in an Iranian city.

## Materials and Methods

### Design and participants 

The study took place in Tabriz city, Iran between March and May, 2017. Tabriz, a city of approximately 1.75 million people in northwestern Iran, has 10 defined zones, each of which includes numerous health centers. Two centers, not geographically contiguous, were randomly designated as the source for either the intervention or non-intervention participants using a random-numbers table. Participants were randomly selected from among family health record profiles.

Inclusion criteria included women 40-60 years of age who were either menopausal (cessation of their menstrual cycle for at least one year) or perimenopausal (women with newly-inconsistent menstrual cycles, heavy menses or menses accompanied by blood clots, and menstrual periods lasting several days longer (>7 days) than had been the woman’s norm.^20,21^ Additional eligibility criteria included being mentally and cognitively able to be interviewed and complete questionnaires, and being physically able to participate in a PA program. Following Moeini et al, mean and standard deviation of PA variables were obtained to calculate effect size. Based on a confidence level of 95% and a power of 80%, required sample size was estimated to be at least 73 women in each group. With this sample size, a difference of about 10% would be required for detection.^22^

### Women’s neighborhood networks

In the intervention cluster, neighborhood networks of women were used, in order to encourage participation in the educational and PA options being offered. The non-intervention group received routine health care.

For the intervention group, four health volunteers from the relevant district health center were invited to participate in identification of existing social networks in their neighborhoods. Health volunteers are local women trained to serve as knowledgeable intermediaries linking health care providers and households. These volunteers had strong relationships with the health care providers and women of the community.

The health volunteers identified 10 women in the community who had good social relationships and effective roles in the social activities of local mosques. These women were invited to participate as mediators and facilitators between health volunteers and menopausal women in the community. Based on the collaboration of health volunteers and mediators/facilitators, health volunteers and facilitators approached women who met the study’s criteria and recruited them into the women’s PA networks.

### Interventional procedures

The intervention group was organized into 18 PA neighborhood groups of five to six women, clusters of five to six women living in close proximity to one another. This enabled them to share information and provide mutual support and encouragement.

All of the 18 neighborhood groups were first invited to educational sessions. Table 1 presents the educational sessions offered. For the educational component and based on the intake questionnaires assessing PA level, the intervention group was divided into two cohorts. Those deemed to be more sedentary were offered an initial five sessions to raise their awareness of the benefits of PA. All intervention group participants were then offered three sessions designed to encourage and organize their participation in PA.

The educational intervention sought to increase participants’ awareness about PA and encourage women to initiate and sustain PA in a gym. The intervention provided participants with knowledge about the appropriate intensity level of PA and how it could be incrementally increased, how to incorporate PA as a natural part of their life, and how to find activities that were pleasant.

After the educational sessions, the members of each neighborhood network group were encouraged to exercise together at a gym. The PA program was designed by a sports medicine specialist, based on the knowledge that a majority of participating women were overweight or obese. The local PA promotion program was named “Healthy Woman, Healthy Family.” Participants were invited for a structured PA program that was held three days a week for three consecutive months under supervision of a coach.

The PA program started at a half-hour daily of low-level activity, progressing to an hour of moderate level activity by the final week, which was proportional to the status and biological age of the target group. The exercise regimen is described in Table 2. Other than completing the initial and final questionnaires, the non-intervention group was offered their normal routine healthcare.

### TTM theoretical variables

We adopted the transtheoretical model (TTM) as a conceptual framework for this study because it has been reported effective in fostering behavioral changes such as PA promotion among women.^23,24^ This model posits that changes in individual health behavior progresses through six stages: pre-contemplation, contemplation, preparation, action, maintenance, and termination. Prochaska and Velicer confirm a “rule of thumb” calculation that for at-risk populations, 40% are in pre-contemplation, 40% are in contemplation, and 20% are in preparation.^25^ Results of a randomized controlled trial support the applicability of TTM for improving PA in women.^26^

In this study, stages of implementing PA were determined by administering the Stages of Exercise Change questionnaire,^27^ which was administered pre- and post-study. Stages of adopting PA were classified according to the following definitions. The *pre-contemplation* stage consisted of women who were not thinking about exercise. Women were in the *contemplation* stage if they had never participated in exercise but were thinking of starting within six months. The *preparation* stage captured those who had an intention to take action in the immediate future (one month) and had a plan of action. Finally, women were defined as being in the *action* stage if they had participated in regular PA within the last six months, and they were coded to the *maintenance* stage if they had engaged in regular PA for more than six months prior to the study.^20^ There were no participants in the *termination* phase.

Decisional balance is part of the core TTM construct. Each person maintains a mental “decisional balance sheet,” which is his or her assessment at any moment in time of the pros and cons of engaging in a specific change of behavior.

Self-efficacy is another core construct of TTM. Built on Bandura’s self-efficacy theory,^28^ this is the confidence people have that they will succeed in their performance of a future task. This is essentially a measure of self-confidence; higher levels of self-efficacy lead to greater changes in behavior.

Self-efficacy was measured pre- and post-study using a six-item instrument that included Likert-based response options ranging from 1 (“Not confident at all”) to 5 (“Extremely confident”). The measure showed a good to excellent level of internal consistency for PA self-efficacy (Cornbrash’s alpha= 0.85) and test-retest reliability over a two-week period (0.90).

### Measurements

All women were visited in their homes and interviewed in order to obtain basic demographic and physical data. All of the measurement tools including socio-demographics and health indices, the Menopause Rating Scale (MRS), and the International PA Questionnaire (IPAQ) were published previously.^20,21^ In addition, systematic and consistent anthropometric measures such as height and weight were collected using a tape, fixed-length headboard, and analog scale (Camry, Germany. Capacity, 160 kg, 1 kg increments ).

Decisional balance was assessed pre- and post-study using a 12-item instrument that included Likert-based response options ranging from 1 (“Don’t agree at all”) to 5 (“Strongly agree”).^24^ Internal consistency of Cornbrash’s alpha=0.89 and test-retest reliability over a two-week period=0.90 indicated a high level of reliability.

Self-efficacy was measured pre- and post-study using a six-item instrument that included Likert-based response options ranging from 1 (“Not confident at all”) to 5 (“Extremely confident”). The measure showed a good to excellent level of internal consistency for PA self-efficacy (Cornbrash’s alpha= 0.85) and test-retest reliability over a two-week period (0.90).

### Statistical analyses

Statistical analyses were performed using IBM SPSS Statistics version 21 (IBM SPSS Statistics, IBM Corp, Armonk, USA). The results are presented as frequency (percent) for qualitative (ordinal) data. The Kolmogorov-Smirnov test was used to check for normal distribution of the data. To compare the two groups for background characteristics and baseline measures, the chi-square test was used for qualitative (ordinal) data. The sign test was used to compare the ordinal variables throughout the study within groups. The Mann-Whitney U test was used to compare the results for ordinal qualitative variables, menopause symptoms, between intervention group and non-intervention group at baseline. Ordinal regression for ordinal variables was used to compare the two groups for the measures at the end of the study, adjusting for baseline values. We used a lack-of-fit test that showed the statistical model is a good fit for data *P* >0.05. Results with two-sided *P* values of <0.05 were considered statistically significant.

## Results

**Figure 1:**
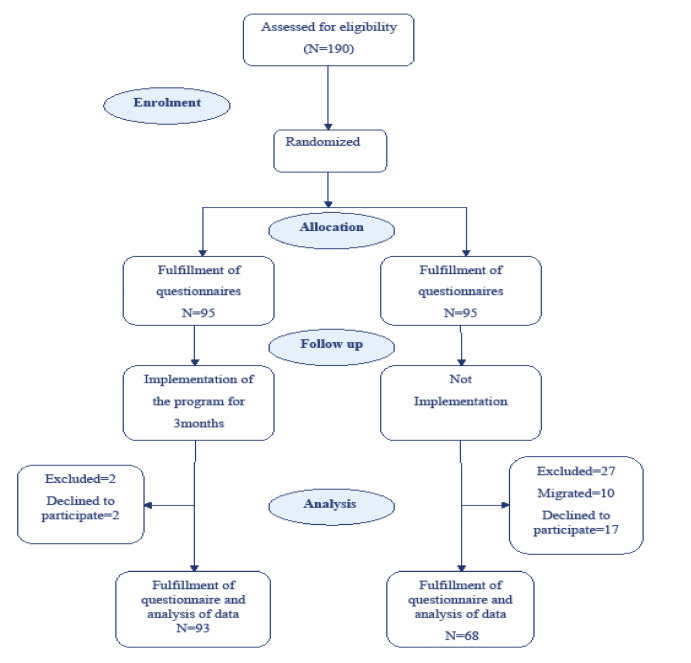
Flow diagram of study

One hundred ninety women were assessed for eligibility; 95 each in the intervention and non-intervention groups. By the time of the final data analysis, the intervention cohort was reduced to 93 by two people declining to participate to completion. The non-intervention group was reduced to 68 through a combination of those declining to participate to completion (n=17) and people moving from their neighborhoods (n=10) (Figure 1). There was no statistically significant difference in basic demographics between the intervention and non-intervention groups (Table 3). None of the participants had a history of MHT use or smoking.

The primary outcome was menopause symptoms and secondary outcome was PA. Menopausal symptom scores (before/after) are presented in Table 4, and PA levels are presented in Table 5. There was no statistically significant difference between the intervention and non-intervention groups at baseline.

At the study’s conclusion, the intervention group showed a significant reduction in the frequency and severity of all menopausal symptoms rated severe or very severe including hot flushes (reduced from 30.1% to 11.8%); sleep problems (declined from 28% to 6.5%); and joint discomfort (reduced from 52.7% to 4.4%) (Table 4). In the non-intervention group, hot flushes, sleep problems and joint problems were significantly worse (*P* < 0.05).

At baseline, 14% (n=13) of the intervention group women were in the action or maintenance stages in regard to PA, and 23.7% (n=22) of the participants were in the preparation stage; 62.3% (n=58) of the women were in the earlier stages (pre-contemplation or contemplation stages) of change. After intervention, all of the participants in the intervention group were in the action or maintenance stages, and their total PA improved (Table 5). For the non-intervention group, the PA stage and total PA remained unchanged after 12 weeks.

In terms of psychological factors, we found that the intervention program significantly improved the PA self-efficacy score from 16 (SD=4.4)) to 23.7 (SD=0.65, *P* < 0.001), while no significant difference was found in PA self-efficacy in the non-intervention group (*P* > 0.05).

The score for decisional balance toward PA increased from 50.24 (SD=6.1) to 59.68 (SD=0.9, *P* < 0.001) in the intervention group while a significant reduction from 48.97 (SD=4.9) to 47.41 (SD=5.5, *P* <0.05) was found in the non-intervention group.

## Discussion

There is an urgent global need for low-cost, accessible, non-pharmacological options to treat menopausal symptoms that affect QOL. This study provided a proof-of-concept test of one such option, which melded educational and social components into a successful program.

The *educational* component of this study provided the information and impetus for women to achieve and sustain higher levels of activity, which provided statistically significant improvement in the menopausal symptoms affecting their QOL. The effectiveness of educational and training programs on improving regular PA has been shown in the previous researches.^1,29^

The *social* component played an important role in sustaining the women’s participation. The 18 neighborhood networks provided a mechanism for continuous interaction that helped raise the awareness of all participants. Awareness raising, in turn, helped women to move from pre-contemplation and contemplation stages to the preparation stage. Neighborhood networks, by providing commitment and re-commitment to act, help initiate movement to the action stage through “self-liberation.”^30^

Neighborhood networks provide what Glanz et al term “dramatic relief,” in which women’s communication with one another about the unhealthy consequences of being sedentary offered emotional motivation and support. Consistent with Rimer and Viswanath’s concepts of social liberation and counterconditioning, the invitation to participate in the gym-based PA program provided a visible alternative for women to learn about a specific strategy that could substitute for being sedentary.^30^

The results of this study showed considerable improvement in PA and a statistically significant reduction in the frequency and severity of menopausal symptoms in the intervention group. Our results provide support for and expansion of prior research that shows the close association between participation in social networks with friends and neighbors and higher PA.^31,32^

The educational program was carried out through group discussion, which has been suggested in the literature as an effective method for helping women change their sedentary habits.^26^ Using the transtheoretical framework, we developed an educational program that was tailored to a specific change segment including women in action and pre-action stages.^29^ The neighborhood networks in this study reflected several of TTM processes that facilitate health education programs.^30^

Some studies^33,34^have suggested that PA has no effect on menopausal vasomotor signs, but most of these studies lacked an educational component.

We also found that women’s decisional balance for performing regular PA and self-efficacy of PA improved after participating in the intervention program. This is consistent with the result from a systematic review that revealed a significant and strong association was between changes in self-efficacy and commitment to engaging in PA.^35^ Some studies have found that self-efficacy is a consistent predictor of the adoption and maintenance of PA.^36,37^

The educational and PA programs provided basic health knowledge and empowered individuals to take personal control over their own health. Specifically, it enabled people to evaluate their own perceived gains and losses associated with PA-related behaviors, thus enabling them to construct an internal decisional balance sheet.^38^ Previous studies showed the important effect of education on self–efficacy improvement and decisional balance.^23,38,39^ It would be interesting to evaluate the impact of neighborhood networks’ development on improvement of self-efficacy as a community based initiative method.

This study has several strengths, one of which is the use of the neighborhood networks as a motivational strategy of PA promotion for reduction of menopause symptoms for the first time. We also reduced the likelihood of confounder bias as possible by increasing similarity of both intervention and control groups with random allocation concealment and adjustment in regression analysis. Data analyzer was also blinded.

This study had a number of limitations. First, social networks were a key component of the strategy. Such networks may form and act in very different ways depending on a number of factors relating to culture, rural vs. urban location, income, class, and other variables. This educational-social strategy for fostering PA engagement would benefit from testing (and modification, where needed) in a wide variety of cultural and demographic settings.

Other limitations included:

- Data collection was based on self-reported questionnaires, which may be subject to recall bias.
- A group that engaged in exercise without educational group discussion was not included in this study, and there was no group that performed exercise independently, without neighborhood network help. A future three-arm randomized controlled trial method would permit evaluation of the efficacy of these options.
- The non-intervention group did not reach and sustain the minimum sample size needed (n=75), which may reduce the observed power of the study.
- Since in behavior change for human samples, it is not possible to blind participations; therefore, in our study, there was a possibility of performance bias due to lack of blinding of interventional group.
- Due to lack of financial support, we measured body mass index (BMI), instead of body fat %.
- Finally, using a non-standard instrument for collecting demographic and health indices information was a limitation of this study. It may have led to misclassification of information and/or lack of comparability to prior studies that have used standard instruments.

## Conclusion

PA, combined with an educational precursor and strong social reinforcement through neighborhood networks, can be a safe, low-cost, effective, and accessible tool for reducing or eliminating many menopausal symptoms. This strategy holds the promise of eliminating economic and geographical barriers to accessing relief for tens of millions of women globally.

## Acknowledgements

We would like to thank Urban Health Center of Tabriz city for its sincere collaboration and all women who participated in this study. We are thankful for Brian Weiss for language editing.

## Funding

No funding.

## Competing interests

The authors state that they have no conflicts of interest.

## Ethics approval

This study was conducted in accordance with the Declaration of Helsinki. The ethics committee at Tabriz University of Medical Sciences (Ethics Code: 923) approved this protocol. Informed written consent was obtained from all the participants before the interviews.

## Authors’ contributions

All authors read and approved the final manuscript. All authors made contributions to conception and design, acquisition of data, or analysis and interpretation of data. All authors analyzed and wrote the manuscript and revised it critically for important intellectual content and edited the manuscript. Finally, all authors agreed to be accountable for all aspects of the work in ensuring that questions related to the accuracy or integrity of any part of the work were appropriately investigated and resolved.

**Table 1:** Health education intervention and processes of change of TTM

**Session**s	**Subjects for sedentary samples about the role of regular physical activity on menopausal symptoms**	**Processes of change that mediate progression between the stages of pre-contemplation, contemplation and preparation**
1	Reducing menopausal symptoms	• Consciousness raising • Environmental reevaluation • Dramatic relief • Self-reevaluation
2	Reducing the risk of chronic diseases	
3	Physical activity decisional balance	
4	Maintain fitness and youthfulness	
5	Introducing successful model in having regular physical activity as well as unsuccessful models	
**Sessions**	**Topics for sedentary as well as active participants**	**Processes of change that mediate progression between the stages of preparation, action and maintenance**
1	Introduction. Proper physical activity and formation of neighborhood networks to promote physical activity	• Self-liberation • Helping relationships • Stimulus control • Counter-conditioning • Reinforcement management
2	Discussion about standard physical activity	
3	Discussion about benefits of group activities such as: • Using a local recreation center • Using group facilitation for registration • Going to weekend physical activities with family • Creating a weekly schedule • Introducing a local club and invitation for that club	

**Table 2:** Physical activity program for menopausal women

**Week(s)**	**Exercises**	**Time (min)**
1	Typical walking with full inhalation and exhalation	5
	Stretching and vibration exercise with upper and lower limbs	5
	Walking at 80 steps/minute	20
	Stretching exercises	5
2	Same as week 1	35
3	Same as week 1 but walking for 25 minutes at 80 steps/min	40
4-5	Same as week 1 but walking for 30 minutes at 90 steps/min	45
6-7	Same as week 1 but walking for 35 minutes at 95 steps/min	50
8-9	Same as week 1 but walking with long strides for 40 minutes at 100 steps/min	55
10-11	Same as weeks 8-9 but walking for 45 minutes	60
12	Same as weeks 10-11	60

**Table 3:** Baseline characteristics of menopausal women

**Variables**	**Intervention** **Group (n=93)**	**Control Group (n=68)**
	**No. (%)**	**No. (%)**
Age		
40-44	19 (20.4)	21 (30.9)
45-50	29 (31.2)	17 (25.0)
51-55	30 (32.3)	18 (26.5)
56-60	15 (16.1)	12 (17.6)
BMI		
Underweight	0	0
Normal weight	8 (8.6)	10 (14.7)
Overweight	42 (45.2)	27 (39.7)
Obese	43 (46.2)	31 (45.6)
Education^a^		
<Primary	12 (12.9)	13 (19.1)
Primary	51 (54.8)	29 (42.6)
Secondary & high school	30 (32.3)	26 (38.2)
Marital status^a^		
Married	84 (90.3)	60 (88.2)
Not married	9 (9.7)	8 (11.6)
Menopause status^a^		
Peri-menopause	48 (51.6)	38 (55.9)
Post-menopause	45 (48.4)	30 (44.1)

Abbreviation: BMI, body mass index. ^a^ Non-significant difference between groups at baseline (P>0.05, chi-square tests).

**Table 4:** Symptoms of intervention and control groups

		**Intervention Group (n=93)**			**Control Group (n=68)**		
**Symptoms**		**Baseline** ^a^ **No. (**%)	**End** ^b^ **No. (%)**	***P*** **value** ^c^	**Baseline** ^a^ **No. (%)**	**End** ^b^ **No. (%)**	***P*** **value** ^c^
Hot Flushes				<0.001			0.052
	None	26 (28.0)	49 (52.7)		30 (44.1)	21 (30.9)	
	Mild	22 (23.7)	22 (23.7)		11 (16.2)	10 (14.7)	
	Moderate	17 (18.3)	11 (11.8)		8 (11.8)	11 (16.2)	
	Severe	11 (11.8)	0		3 (4.4)	10 (14.7)	
	Very severe	17 (18.3)	11 (11.8)		16 (23.5)	16 (23.5)	
Heart Discomfort				<0.001			0.041
	None	46 (49.5)	68 (73.1)		49 (72.1)	42 (61.8)	
	Mild	19 (20.4)	14 (15.1)		5 (7.4)	12 (17.6)	
	Moderate	10 (10.8)	6 (6.5)		5 (7.4)	4 (5.9)	
	Severe	14 (15.1)	3 (3.2)		4 (5.9)	5 (7.4)	
	Very severe	4 (4.3)	2 (2.2)		5 (7.4)	5 (7.4)	
Sleep Problems				<0.001			0.002
	None	40 (43.0)	67 (72.0)		42 (61.8)	31 (45.6)	
	Mild	11 (11.8)	16 (17.2)		7 (10.3)	6 (8.8)	
	Moderate	16 (17.2)	4 (4.3)		0	6 (8.8)	
	Severe	13 (14.0)	2 (2.2)		9 (13.2)	7 (10.3)	
	Very severe	13 (14.0)	4 (4.3)		10 (14.7)	18 (26.5)	
Depressive Mood				<0.001			0.596
	None	37 (39.8)	66 (71.0)		33 (48.5)	26 (38.2)	
	Mild	10 (10.8)	17 (18.3)		10 (14.7)	17 (25.0)	
	Moderate	21 (22.6)	6 (6.5)		9 (13.2)	11 (16.2)	
	Severe	16 (17.2)	1 (1.1)		5 (7.4)	5 (7.4)	
	Very severe	9 (9.7)	3 (3.2)		11 (16.2)	9 (13.2)	
Irritability				<0.001			0.310
	None	36 (38.7)	60 (64.5)		38 (55.9)	34 (50.0)	
	Mild	11 (11.8)	24 (25.8)		9 (13.2)	7 (10.3)	
	Moderate	18 (19.4)	4 (4.3)		5 (7.4)	13 (19.1)	
	Severe	20 (21.5)	4 (4.3)		7 (10.3)	7 (10.3)	
	Very severe	8 (8.6)	1 (1.1)		9 (13.2)	7 (10.3)	
Anxiety				<0.001			0.188
	None	33 (35.5)	55 (59.1)		36 (52.9)	34 (50.0)	
	Mild	14 (15.1)	25 (26.9)		7 (10.3)	7 (10.3)	
	Moderate	19 (20.4)	7 (7.5)		5 (7.4)	13 (19.1)	
	Severe	17 (18.3)	4 (4.3)		10 (14.7)	7 (10.3)	
	Very severe	10 (10.8)	2 (2.2)		10 (14.7)	7 (10.3)	
Exhaustion				<0.001			0.136
	None	13 (14.0)	50 (53.8)		19 (27.9)	13 (19.1)	
	Mild	15 (16.1)	29 (31.2)		10 (14.7)	6 (8.8)	
	Moderate	19 (20.4)	6 (6.5)		9 (13.2)	17 (25.0)	
	Severe	26 (28.0)	3 (3.2)		10 (14.7)	12 (17.6)	
	Very severe	20 (21.5)	5 (5.4)		20 (29.4)	20 (29.4)	
Sexual Problems				<0.001			0.078
	None	31 (33.3)	41 (44.1)		33 (48.5)	23 (33.8)	
	Mild	6 (6.5)	16 (17.2)		3 (4.4)	6 (8.8)	
	Moderate	14 (15.1)	9 (9.7)		6 (8.8)	4 (5.6)	
	Severe	18 (19.4)	15 (16.1)		8 (11.8)	15 (22.1)	
	Very severe	24 (25.8)	12 (12.9)		17 (25.0)	20 (29.4)	
Bladder Problems				<0.001			0.151
	None	60 (64.5)	74 (79.6)		48 (70.6)	39 (57.4)	
	Mild	10 (10.8)	10 (10.8)		2 (2.9)	9 (13.2)	
	Moderate	11 (11.8)	4 (4.3)		8 (11.8)	9 (13.2)	
	Severe	8 (8.6)	4 (4.3)		4 (5.9)	8 (11.8)	
	Very severe	4 (4.3)	1 (1.1)		6 (8.8)	3 (4.4)	
Vaginal Dryness				<0.001			0.189
	None	55 (59.1)	67 (75.3)	54 (79.4)	45 (66.2)
	Mild	4 (4.3)	10 (11.2)	2 (2.9)	8 (11.8)
	Moderate	13 (14.0)	6 (6.7)	3 (4.4)	3 (4.4)
	Severe	12 (12.9)	4 (4.5)	3 (4.4)	5 (7.4)
	Very severe	9 (10.3)	2 (9.7)	6 (8.8)	7 (10.3)
Joint Discomfort				<0.001			0.049
	None	24 (25.8)	46 (49.5)		23 (33.8)	11 (16.2)	
	Mild	5 (5.4)	36 (38.7)		6 (8.8)	13 (19.1)	
	Moderate	15 (16.1)	7 (7.5)		12 (17.6)	11 (16.2)	
	Severe	22 (23.7)	2 (2.2)		11 (16.2)	14 (20.6)	
	Very severe	27 (29.0)	2 (2.2)		16 (23.5)	19 (27.9)	

^a^ Non-significant difference between groups at baseline (P>0.05, Mann-Whitney U). ^b^ Significant difference between intervention and control groups after intervention (*P*  < 0.001ordinal regression adjusted for baseline measurements) ^c^ Sign test. Percentages may not add to 100% due to rounding error.

**Table 5:** Physical activity of intervention and control groups

**Physical Activity** **(MET-min/ week)** ^a^	**Intervention** **(n=93)**	**Control** **(n=68)**	***P*** **value** ^b^
**Baseline**			
Low (<600)	18 (19.4)	14 (20.6)	0.980
Moderate (600-3000)	58 (62.4)	42 (61.8)	
Extreme (>3000)	17 (18.3)	12 (17.6)	
**Final Outcome**			
Low (<600)	0	17 (25.0)	<0.001
Moderate (600-3000)	53 (57.0)	43 (63.2)	
Extreme (>3000)	40 (43.0)	8 (11.8)	
*P* value	<0.001^c^	0.251^d^		

^a^ METs (metabolic equivalents) ^b^ Chi-square test. ^c^ Non-significant difference between groups at baseline (P>0.05, Mann-Whitney U). ^d^ Significant difference between intervention and control groups after intervention (P < 0.001ordinal regression adjusted for baseline measurements).
